# Potential of a polyherbal drug to prevent antimicrobial resistance in bacteria to antibiotics

**DOI:** 10.1038/s41598-018-28966-x

**Published:** 2018-07-18

**Authors:** Tapas Kumar Sar, Indranil Samanta, Achintya Mahanti, Shabnam Akhtar, Jeevan Ranjan Dash

**Affiliations:** 1 Department of Veterinary Pharmacology and Toxicology, West Bengal University of Animal and Fishery Sciences, 37, K.B. Sarani, Kolkata, 700037 WB India; 2 Department of Veterinary Microbiology, West Bengal University of Animal and Fishery Sciences, 37, K.B. Sarani, Kolkata, 700037 WB India

## Abstract

Persistence of antibacterial drugs for prolonged period in milk increases the probability of antimicrobial resistance progress. Ceftizoxime was found to be excreted in milk for a prolonged period in goats, cows and buffaloes following intravenous injection of ceftriaxone and ceftizoxime. A single dose of ceftriaxone was administered intravenously in healthy control goats (group I) and a single oral dose of the commercial mammary protective polyherbal drug (1.9 gm) was given one hour prior to intravenous ceftriaxone injection in healthy (group II) and induced mastitic (group III) goats to evaluate milk disposition of ceftizoxime following single intravenous dosing of ceftriaxone at 42.25 mg kg^−1^.Ceftriaxone/ceftizoxime was analyzed by HPLC. The t_1/2_α and t_1/2_β values were 14.755 ± 2.733 and 149.079 ± 18.565 hour, respectively indicating prolonged persistence of ceftizoxime in milk. The polyherbal drug increased the milk concentration at later hours and hastened the excretion of ceftizoxime from milk compared to control group. Ceftriaxone could not be detected in milk. The study suggested that adjunct single or repeated therapy of  the polyherbal drug may cause non persistence of ceftriaxone and shorter persistence of ceftizoxime in milk.

## Introduction

Mastitis, a menace with serious concern in dairy industry, is responsible for significant economic losses due to reduced milk yield (up to70%), milk discard after treatment (9%), cost of veterinary services (7%) and premature culling (14%)^1^. Apart from its economic importance, it also carries public health significance^2^. Ceftriaxone, a third generation cephalosporin, is active against a wide range of gram negative and gram positive organisms and is used more frequently for the treatment of lower respiratory tract infections, urinary tract infections, peritonitis, skin and soft tissue infections and septicemia caused by sensitive organisms both in human and veterinary practices. The commercial polyherbal drug (Fibrosin^®^) facilitates cleaning of udder by clearing the tissue debris and aids in down flow of milk during mastitis (leaflet of Fibrosin^®^, Legend Remedies Pvt. Ltd.). However, the manufacturing company could not claim or report the new action of the polyherbal drug (Fibrosin^®^) presented in this article. Fibrosin^®^ enhanced the penetration of ceftizoxime through milk-blood barrier and increased the bioavailability of ceftizoxime in milk following a single dose intracisternal administration of ceftriaxone after its metabolism in liver^3^. The polyherbal drug also returned increased milk alkaline phosphatase and catalase activity in mastitic goats to normal level and maintained normal glutathione level and significantly increased lactoperoxidase activity with 1 hr prior oral administration to single intravenous dosing of ceftriaxone^4^. Mastitis is an inflammatory condition of the mammary gland, frequently caused by bacteria. Selection of the antimicrobial agent and maintenance of therapeutic concentration of the drug at the site of infection determine the success rate of antibiotic therapy in mastitis. Ceftriaxone was found to be converted to a major active metabolite i.e. ceftizoxime (another third generation cephalosporin) after induction of cytochrome P_450_ in liver following a single dose intravenous administration that persisted for a longer period in milk of goats^5^. Primarily ceftizoxime, not ceftriaxone penetrated inflamed mammary gland of mastitic cows at a higher degree^6^. Ceftizoxime, a broad spectrum antibiotic is effective against a wide variety of aerobic/anaerobic gram positive and gram negative bacteria and remains highly stable in presence of β lactamases. It was reported that a lower concentration of ceftriaxone is excreted in milk of goats following single dose intravenous and intramuscular administration at 20 mg kg^−1^ body weight^7^. However, ceftizoxime was detected in milk at an appreciable level for 720 hr and 24 hr post dosing, respectively following intravenous and intramuscular dosing of ceftriaxone at 50 mg kg^−1^ in lactating goats^5,8^. Longer persistence of ceftizoxime in milk (at least 96 hr) of goat and cow following a single parenteral injection starting from early time of collection is a clear indication for intravenous administration of ceftriaxone particularly at a higher dose rate^5,6^. Though antibiotic can kill bacteria but cannot prevent mammary gland tissue damage. But, the polyherbal drug was found to prevent tissue damage by producing antioxidant activities^4,8,9^. Milk can be considered as a secretion cum excretion of the mammary gland. Therefore, it can be expected that the polyherbal drug will enhance excretion of ceftizoxime from milk compartment shortening the persistence of eftizoxime residue in milk after achieving higher concentration for a certain period of time during the late hours following single intravenous dosing of ceftriaxone. Though the pharmacokinetic study of ceftriaxone was conducted in many ruminant species following parenteral administration, e.g., sheep^10^ cow calves^11^, crossbred calves^12,13^, buffalo calves^14,15^, lactating goats^3,5,7^ and crossbred mastitic cows^6^ but disposition study of its active metabolite, e.g., ceftizoxime to determine pharmacokinetic parameters in milk following intravenous dosing of the parent ceftriaxone was not done. Dosing interval of antibacterial drug in mastitis or other bacterial infections also should be based on maintenance of therapeutic concentration of its active metabolite at the site of infection e.g., mammary gland/milk in mastitis particularly when the metabolite is the major active metabolite of the parent drug and when the active metabolite, not the parent drug excretes through milk at a higher rate. Persistence of antibacterial drugs for longer period in milk increases chances of development of antimicrobial resistance^16^. Therefore, the present study was conducted to explore disposition of ceftizoxime in milk of healthy lactating and mastitis (experimental models) in goats following single intravenous dosing of ceftriaxone without and with 1 hr prior oral administration of mammary gland protective herbal drug (Fibrosin^®^) with another aim to evaluate whether the polyherbal can help to conserve both the antibiotics as effective one.

## Methods

### Drugs

The drugs used in this study were ceftriaxone (Aristo Pharmaceuticals, India), ceftizoxime (GlaxoSmithKline Pharmaceuticals Ltd., Nashik, India) and Fibrosin^®^ (Legend Remedies Pvt. Ltd., Vadodara, India). Fibrosin^®^ is composed of Kanchanar-gugal (gummy substances and resins of *Bauhinia variegata* Linn.), Chitrak-mula (root of *Plumbago zeylanica*), Punar-navastaka (flower of *Triaanthema monogyna*), Trifala (fruit of *Terminalia belerica* Retz. + fruit of *Terminalia chebula* Retz. + fruit of *Phylanthus amblica*) and Apamarga (whole plant of *Achyranthes aspera* Linn.). The actual composition of Fibrosin^®^ is trade secret of Legend Remedies Pvt. Ltd., Vadodara, India.

### Animals

Apparently healthy lactating black Bengal goats (1.5 to 2 years of age) weighing 11.830–14.200 kg were considered for this study. A total of 18 lactating goats were used for this experiment. The animals were divided in three groups each containing 6 goats. The animals were caged individually in custom-made, stainless steel cages and standard feed ration was provided. The temperature of the experimental animal room was maintained at 22 °C (±3 °C) and artificial lighting facilities were provided. Before the start of the experiment, the animals were acclimatized for 7 days.

## Experimental design

The goats produced milk containing negligible quantity of *Staphylococcus aureus* were included in this study. Mastitis was induced in group III goats as per our established method^17^. *Staphylococcus aureus* (strain J638) was isolated from the mastitic milk sample from a Jamunapuri goat in mannitol salt agar (HiMedia, India), which was confirmed by colony characteristics in MSA, Gram-staining and standard biochemical tests such as catalase, oxidase, indole, Methyl Red, Voges-Proskauer, urease, carbohydrate fermentation, and coagulase test^18^. The isolate was found resistant against penicillin G, ampicillin, amoxicillin and tetracycline. Twelve clinically healthy lactating female black Bengal goats after 21^st^ day of parturition were inoculated with 36,000 CFU of locally isolated coagulase positive *S*. *aureus* strain (J638) by sterile intramammary infusion tube. The inoculated animals were not milked out during the first 3 days post-inoculation. The animals were closely observed for the development of any clinical sign for 4 weeks.

The group-I lactating goats were considered as control group and a single dose of only ceftriaxone at 42.25 mg kg^−1^ body weight was administered intravenously. In group-II healthy and group-III mastitic goats, a single dose of Fibrosin® (1.9 gm) mixing in 100 ml distilled water was administered orally 1 hr prior to the intravenous injection of ceftriaxone at the same dose rate. The polyherbal drug was orally administered in empty stomach of all goats of group II and III to avoid any feed interaction. A single intravenous dose of ceftriaxone at 42.25 mg kg^−1^ was administered to the lactating goats without and with 1 hr pre single dose (1.9 gm) oral administration of the polyherbal drug (Fibrosin^®^) in the present study needed no permission from Institutional Animal Ethics Committee because we previously administered ceftriaxone at 50 mg kg^−1^ intravenously to lactating goats with and without a single oral dose (1.9 gm) of Fibrosin^®^.

### Collection of milk samples

The milk samples (1 ml) were collected aseptically from both teats into the test tubes and 1 ml was taken from the pooled sample. Milk samples were collected at 0 and at 0.08, 0.16, 0.25, 0.33, 0.50, 0.66, 1, 3, 4, 6, 8, 12, 24, 36, 48, 72, 96, 120, 144, 168, 192, 216, 240, 288, 336, 360, 480, 576, 600, and 720 hr post-dosing.

### Analytical methods

Ceftizoxime concentrations were analyzed by high performance liquid chromatography (HPLC) from milk by the reported method^3^ and confirmed^5^. The limit of detection for both the drugs was 0.01 µg ml^−1^. The retention times of ceftriaxone and ceftizoxime were 9.654 and 3.393, respectively which showed inter-day variations.

The phenols along with flavonoids from Fibrosin^®^ were estimated by our team according to method^19^. The tannins were also estimated as per method^20^ from Fibrosin^®^.

### Pharmacokinetic analysis

The Pharmacokinetic parameters of ceftizoxime in milk in term of the parent compound were calculated^21^ using the effective ratio.

$${\rm{Effective}}\,{\rm{ratio}}=\frac{{\rm{molecular}}\,{\rm{weight}}\,{\rm{of}}\,{\rm{metabolite}}}{{\rm{Molecular}}\,{\rm{weight}}\,{\rm{of}}\,{\rm{parent}}\,{\rm{drug}}}$$

$${\rm{Concentration}}\,{\rm{of}}\,{\rm{parent}}\,{\rm{drug}}=\frac{{\rm{concentration}}\,{\rm{of}}\,{\rm{metabolite}}}{{\rm{Effective}}\,{\rm{ratio}}}$$

Disposition kinetic parameters of ceftizoxime in milk were determined according to the method^22^.

### Statistical analysis

The data were analyzed by paired *t*-test and *t*-test of independent sample assuming equal variance using SPSS 10.0 (Manufactured by SPSS Inc., USA).

## Results

### Recovery of ceftizoxime

**Figure 1:**
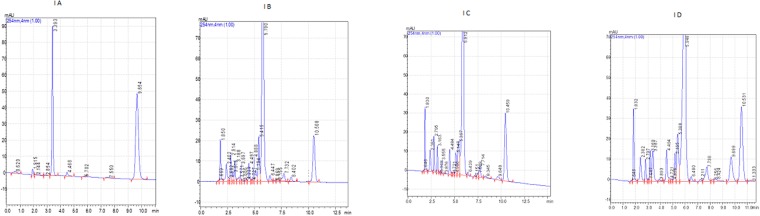
(**A**) Chromatogram of ceftriaxone (20 ppm) and ceftizoxime (20 ppm) (**B**) Chromatogram of milk (blank sample) (**C**) Chromatogram of ceftriaxone (0.5 ppm) and ceftizoxime (0.5 ppm) in milk (**D**) Chromatogram of ceftriaxone (10 ppm) and ceftizoxime (10 ppm) in milk.

The recovery percentage of ceftizoxime was 80.37 ± 2.30% in milk and the limit of detection for ceftizoxime in milk was 0.01 ppm. The retention time of ceftriaxone and ceftizoxime were respectively 9.654 and 3.393 (Fig. 1).

### Quantification of few active principles

The aqueous solution of Fibrosin (4 gm dissolved in 100 ml distilled water) was found to contain phenols (0.054 gm gallic acid equivalent/gm of Fibrosin dry bolus), flavonoids (0.26 g quercetin equivalent/gm of Fibrosin dry bolus) and tannins (0.142 gm tannic acid equivalent/gm of Fibrosin dry bolus). The ethanolic extract of Fibrosin (4 gm dissolved in 100 ml distilled water) was found to contain phenols (0.02 gm gallic acid equivalent/g of Fibrosin dry bolus), flavonoids (0.075 g quercetin equivalent/gm of Fibrosin dry bolus) and tannins (0.065 gm tannic acid equivalent/gm of Fibrosin dry bolus).

### Milk level of ceftizoxime

The milk concentration of ceftizoxime was gradually increased with advancement of time, peaked at 72 hr post-dosing (383.20 ± 19.53 µgm/ml) in healthy lactating goats and then declined bit by bit persisting up to 720 hr post dosing (Table 1). In group-II fibrosin treated healthy goats, milk concentration of ceftizoxime followed zigzag pattern which showed significantly higher concentration of the drug from 216 hr to 360 hr post-dosing compared to group-I and group- III goats. Ceftizoxime in milk could not be detected at 480 hr post dosing in group-II goats. The milk concentrations of ceftizoxime in group-III fibrosin treated mastitic goats were decreased significantly at different time compared to group-I healthy goats without fibrosin treatment but persisted at a significantly higher concentrations at 312 and 336 hr post dosing compared to group-I healthy goats. However, ceftizoxime in milk could not be detected at 360 hr post dosing in these mastitic goats. All the mastitic goats recovered completely from all the clinical signs on 120 hr post dosing.

**Table 1:** Mean milk concentration (µg ml^−1^) of ceftizoxime in healthy goats and with 1 hour prior single oral dosing of Fibrosin® (1.9 g) in healthy and mastitic goats following single dose intravenous administration of ceftriaxone at 42.25 mg kg^−1^.

Time (hr)	Gr-I (healthy)	Gr-II (healthy + fibrosin)	Gr-III (mastitis + fibrosin)
0.08	1.71^a^ ± 0.25	24.12^b^ ± 3.05	Agalactia
0.16	2.64^a^ ± 0.43	3.17^ab^ ± 0.42	Agalactia
0.25	5.23^a^ ± 0.56	22.33^b^ ± 1.37	Agalactia
0.33	7.17^a^ ± 0.85	3.34^b^ ± 0.45	Agalactia
0.5	21.13^a^ ± 1.95	25.78^b^ ± 1.52	Agalactia
0.66	34.23^a^ ± 2.59	47.92^b^ ± 2.79	Agalactia
1	37.38^b^ ± 2.55	3.62^a^ ± 0.93	Agalactia
2	41.28^b^ ± 1.32	4.65^a^ ± 1.28	Agalactia
3	46.22^ab^ ± 1.73	42.31^a^ ± 3.28	Agalactia
4	72.14^a^ ± 7.71	73.23^ab^ ± 5.26	Agalactia
6	122.76^a^ ± 6.54	138.23^ab^ ± 10.51	Agalactia
8	184.32^b^ ± 14.79	208.78^bc^ ± 18.16	65.06^a^ ± 6.27
12	223.66^b^ ± 14.59	265.45^bc^ ± 19.45	98.12^a^ ± 6.18
24	256.27^c^ ± 19.56	153.43^a^ ± 16.04	159.87^ab^ ± 7.93
36	350.66^b^ ± 18.62	367.87^bc^ ± 28.13	122.32^a^ ± 6.01
48	365.53^c^ ± 22.59	107.67^a^ ± 8.36	132.87^ab^ ± 6.47
72	383.20^c^ ± 19.53	245.76^b^ ± 23.26	44.34^a^ ± 5.63
96	340.87^c^ ± 17.65	236.78^b^ ± 7.43	21.05^a^ ± 2.37
120	288.87^c^ ± 7.91	111.22^a^ ± 8.02	124.23^ab^ ± 7.21
144	256.33^c^ ± 10.96	85.08^a^ ± 12.95	202.11^b^ ± 11.89
168	232.89^c^ ± 11.25	205.67^bc^ ± 10.44	127.21^a^ ± 6.94
192	218.35^ab^ ± 12.69	254.34^bc^ ± 13.67	207.77^a^ ± 12.16
216	199.08^b^ ± 11.93	238.12^c^ ± 8.62	196.56^ab^ ± 13.96
240	171.33^a^ ± 9.62	253.44^c^ ± 16.96	210.10^ab^ ± 14.51
312	130.65^a^ ± 12.02	268.77^c^ ± 20.91	180.91^b^ ± 13.71
336	112.13^a^ ± 11.59	264.03^c^ ± 21.08	186.85^b^ ± 16.92
360	98.61^a^ ± 11.07	332.78^b^ ± 18.43	BDL (below detectable level)
480	43.50^a^ ± 8.39	BDL	BDL
576	25.29^a^ ± 6.71	BDL	BDL
600	21.03^a^ ± 5.69	BDL	BDL
720	10.67^a^ ± 3.32	BDL	BDL

a, b and c indicate significant difference.

### Kinetic parameters

**Figure 2:**
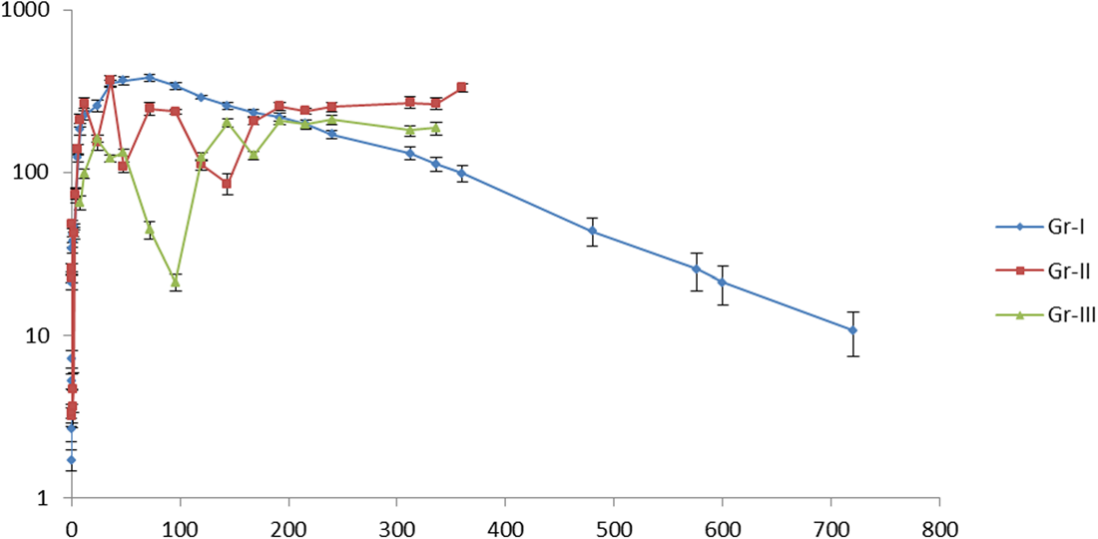
Semi logarithmic plot of mean milk concentration of ceftizoxime (µg ml^−1^) in healthy and mastitic goats following single dose intravenous administration of ceftriaxone at 42.25 mg kg^−1^ without and with 1 hr prior oral administration of Fibrosin^®^ (Y- axis represents concentration and X-axis represents time).

Ceftizoxime followed “two-compartment open model” in milk of healthy lactating goats following single intravenous dosing of ceftriaxone at 42.25 mg kg^−1^ (Fig. 2). The kinetic parameters of ceftizoxime in milk of healthy goats were calculated as described earlier^16,17^ and presented in Table 1. The t_1/2_α and t_1/2_β values were 14.755 ± 2.733 and 149.079 ± 18.565 hr, respectively indicating longer persistence of ceftizoxime in milk. The mean AUC and Vd_area_ values were 100206.30 ± 6824.504 µg hr ml^−1^ and 0.00683 ± 0.00047 lit kg^−1^, respectively indicating wide coverage but comparatively lower distribution of ceftizoxime in the milk compartment. The mean CL_M_ value of 4.48 × 10^−5^ ± 0.909 × 10^−5^ lit kg^−1^ hr^−1^ with higher t_1/2_β value indicated poor clearance of the drug from milk of healthy goats (Table 2).

**Table 2:** Mean kinetic parameters of ceftizoxime in milk of healthy goats following single dose intravenous of ceftriaxone at 42.25 mg kg^−1^.

Kinetic Parameters	Gr –I (healthy goats)
${{\rm{C}}}_{{\rm{M}}}^{0}$ (µg ml^−1^)	1046.108 ± 119.031
α (hr^−1^)	0.054 ± 0.008
β (hr^−1)^	0.005 ± 0.00057
t_1/2_α (hr)	14.755 ± 2.733
t_1/2_β (hr)	149.079 ± 18.565
AUC (µg hr ml^−1^)	100206.30 ± 6824.54
MRT (hr)	300.923 ± 89.273
CL_M_ (L kg^−1^ hr^−1^)	4.48 × 10^−^ ± 0.909 × 10^−5^
Vd_area_ (L kg^−1^)	0.00683 ± 0.00047
C_max_ (µg ml^−1^)	404.831 ± 24.107

${{\rm{C}}}_{{\rm{M}}}^{0}$, theoretical zero time milk drug concentration; α, rate constant related to slope of absorption curve; β, rate constant related to slope of elimination curve; t½ α and t½ β, half-lives of the drug in absorption and elimination phases, respectively; AUC, total area under the milk drug concentration versus time curve from 0 to t α after administration of a single dose; MRT, minimum residential time; Cl_M_: total mammary gland clearance of drug; Vd_area_, apparent volume of distribution of the drug on the total area under milk drug concentration versus time curve; C_max,_ maximum milk drugs concentration.

## Discussion

Ceftrixone having a molecular weight 554.59 is liable to excrete through bile that may undergo absorption in the intestine resulting in its entry into the systemic circulation again followed by metabolism in liver^3,23^. Ceftriaxone showed major hepatic clearence in goats which was evidenced by its higher Cl_H_ value^23,24^. Being a lipid soluble drug, ceftriaxone is expected to be absorbed in presence of bile from the intestine. Failure of bile excretion due to hepatic damage was responsible either for accumulation of drug in the liver or interference with the further absorption of ceftriaxone resulting in absence of reabsorption phase in the disposition of the drug in hepatopathic goats^24^. However, reabsorption of ceftriaxone could not be deteted also in goats following single dose intravenous administration of only ceftriaxone at 50 mg kg^−1^ (a higher dose) as well as at 42.25 mg kg^−1^ and in cows at lower dose rate also^6^ which might be due to optimum induction of cytochrome P_450_ in liver by ceftriaxone with subsequent rapid metabolism as a result of cent percent bioavailability. The mean values of t_1/2_β and AUC also suggested a longer persistence of ceftizoxime in milk. The kinetic parameters of ceftizoxime in milk could not be calculated in group-II healthy and group-III mastitic goats as the drug showed a zigzag pattern in presence of the polyherbal drug. However, ceftizoxime showed uniformity in dispositional pattern starting from 168 hr post dosing to 360 hr post dosing in milk of healthy goats and from 168 hr post dosing to 336 hr post dosing in milk of mastitic goats in presence of the polyherbal drug indicating that the inflammation of mammary gland (mastitis) after a certain period may not influence the effect of the polyharbal drug on disposition of ceftizoxime in milk. In the present study, milk concentration of ceftizoxime was gradually increased with advancement of time, peaked at 72 hr post dosing and then declined bit by bit persisting up to 720 hr post dosing in healthy lactating goats following single intravenous dosing of ceftriaxone at 42.25 mg kg^−1^. Milk concentration of ceftizoxime started to increase from 0.5 hr, achieved its peak level at 12 hr followed by a slow decline in concentration which came to its minimum concentration at 96 hr post dosing in healthy lactating goats after intravenous administration of ceftizoxime at 10 mg kg^−1^ ^25^. The difference of observed data between previous work and the present one may be attributed towards induction of cytochrome P_450_ by ceftriaxone in liver at the higher dose rate of ceftriaxone used in the present study. Hepatic clearance of ceftriaxone predominates renal clearance in goats^23,24^. Ceftriaxone was found to be an inducer of microsomal cytochrome P_450_ in liver and undergoes hydrolysis to produce its active metabolite i.e. ceftizoxime through cleavage of thioether bond present in ceftriaxone^5^.Furthermore, ceftrixone having a molecular weight 554.59 is liable to excrete through bile that may undergo absorption in the intestine resulting in its entry into the systemic circulation again followed by metabolism in liver^3,23,24^. Ceftizoxime in milk at a concentration of 380.16 ± 46.18 µg ml^−1^ at 72 hr post dosing was also detected which gradually declined in concentration to 360 hr with a concentration of 50.50 ± 17.32 µg ml^−1^ in healthy goats following single intramammary dosing of ceftriaxone at 50 mg kg^−1^ with 1 hr prior oral dosing of Fibrosin^®^ (the polyherbal drug)^3^. Disposition of ceftizoxime in milk was best fitted to “two-compartment open model” in group-I healthy lactating goats following single intravenous dosing of ceftriaxone. However, disposition of ceftizoxime showed zigzag pattern in milk of group-II healthy and group-III mastitic goats in presence of the polyherbal drug following single intravenous dosing of ceftriaxone. Interestingly, disposition pattern of ceftizoxime in milk of group-II healthy and group-III mastitic animals, showed uniformity starting from 168 hr post dosing in presence of the polyherbal drug which indicated that mastitis may not influence the effect of the polyherbal drug on disposition of ceftizoxime particularly in milk. Significantly higher concentrations of the drug were detected in milk of group –II fibrosin treated healthy goats from 216 hr to 360 hr post-dosing compared to group-I healthy and group- III fibrosin treated mastitic goats. Significantly higher concentrations of ceftizoxime were also determined in group-III fibrosin treated mastic goats at 312 and 336 hr post dosing compared to group-I healthy goats. These findings suggested that the polyherbal drug enhanced bioavailability of ceftizoxime in milk of both healthy and mastitic goats at late hours of collection and also hastened clearance excretion of the drug from the milk compartment. The polyherbal drug (Fibrosin^®^) is composed of some medicinal plants those contain some active principles which have bioavailability enhancing property. The presence of flavonoids particularly quercetin (a bioenhancer) at a considerable amount may be responsible for increasing concentration of ceftizoxime in milk at latter hours. Oral administration of stem bark powder of *Bauhinia variegata* L. (a plant used in Fibrosin^®^) and leaf juice of *Ocimum sanctum* caused increase in bioavailability of ceftriaxone and ceftizoxime in both plasma and milk of chronic mastitic goats^17,26^. Chitrak-mula (*Plumbago zeylanica*) contained the active principle that also plays as bioenhancer^3^. The polyherbal drug not only increased the bioavailability of ceftizoxime in milk, it also helped to eliminate the drug at a higher degree particularly at late hours resulting in non-availabality of ceftizoxime in milk of both healthy and mastitic goats compared to longer persistence of ceftizoxime in milk of healthy goats without the polyherbal treatment. The polyherbal drug showed both way advantages for treating mastitis with single intravenous dosing of ceftriaxone firstly by increasing the bioavailability of its active metabolite i.e. ceftizoxime in milk of mastitic goats and secondly by reducing the persistence of cetizoxime residue in milk not only in mastitic goats but after parenteral administration of ceftriaxone or ceftizoxime in lactating goats that may subside public health hazard. The mean t_1/2_β value of ceftizoxime in milk of healthy goats was 149.079 ± 18.565 hr following single intravenous dosing of only ceftriaxone at 42.25 mg kg^−1^. A mean t_1/2_β value (87.00 ± 13.80 hr) of ceftizoxime was recorded in milk of healthy goats following single intracisternal administration of ceftriaxone at 50 mg kg^−1^ with 1 hr prior oral dosing of the polyherbal drug (Fibrosin®)^3^. The polyherbal drug also caused shorter persistence of ceftizoxime (active metabolite of ceftriaxone) in milk of both healthy and mastitic goats in our previous studies compared to goats without polyherbal treatment following intravenous administration of ceftriaxone^5^. The mean AUC and Vd_area_ values of ceftizoxime in milk were found to be 100206.30 ± 6824.504 µg hr^−1^ml^−1^ and 0.00683 ± 0.00047 lit kg^−1^, respectively in healthy goats in the present study. The mean AUC and Vd_area_ values of ceftizoxime were reported as 98292.07 ± 17191.48 µg hr^−1^ml^−1^ and 0.006 ± 0.0003 lit kg^−1^ in milk of healthy goats following single intramammary dosing of ceftriaxone at 50 mg kg^−1^ with 1 hr prior oral administration of Fibrosin^®3^ which were more or less similar to our present findings. The mean CL_M_ value of ceftizoxime in milk was observed to be 4.48 × 10^−5^ ± 0.909 × 10^−5^ lit kg^−1^ hr^−1^ in healthy goats without the polyherbal treatment following single intravenous dosing of ceftriaxone at 42.25 mg kg^−1^ in the present study strongly suggested longer elimination of ceftizoxime in milk. The polyherbal drug enhanced the elimination of ceftizoxime (molecular weight- 360.41) at latter hours from milk abridging the persistence of milk residue and reducing associated public health hazard. Third generation cephalosporins like ceftriaxone and ceftizoxime are recommended to treat bacterial infections such as clinical mastitis in cattle, buffalo, sheep and goats^27–29^. Moreover, dosing interval of an antibacterial drug in mastitis should be based on dispositional profile and persistence of its major active metabolite in milk when the active metabolite primarily excretes through milk or it may lead to accumulation of the drug in the mammary gland or milk as ceftriaxone/ceftizoxime is often used to treat bacterial mastitis in livestock. The study was also important to know the clearance of an active drug that is eliminated for a prolonged period in milk when the parent one is given by intravenous route to treat any infectious disease particularly for antimicrobial drug. The elimination of ceftizoxime from milk occurs for a longer period which can be recommended to treat bacterial infections of mammary gland particularly with single intravenous ceftriaxone injection as the drug has the potentiality to prevent recurrence or relapse of bacterial infections in the milk compartment of the mammary gland. Dosing interval of an antibacterial drug in mastitis should be based on dispositional behavior and persistence of its major active metabolite in milk when the active metabolite primarily excretes through milk.

## Conclusion

The studied polyherbal drug (Fibrosin®) can enhance the elimination of ceftizoxime from milk which consequently reduces its persistence in the secreted mastitic milk. As development of antibiotic resistance is dependent on persistence of antibiotic for prolonged period in the milk, the outcome of the present study may be a used as a strategy for reducing the development of antimicrobial resistance. In a similar kind of experiment earlier it was observed that mean duration of treatment for pneumonia with antibiotics was reduced significantly with the use of Chisan®, a polyherbal drug^30^. Moreover, dosing interval of an antibacterial drug in mastitis should be based on dispositional profile and persistence of its major active metabolite in milk when the active antibacterial metabolite primarily excretes through milk. The polyherbal drug may be used as a supportive therapy with both the antibiotics when administered parenterally to treat sensitive bacterial infections in goats, cows and buffaloes whose milk is consumed widely by the people to minimize development of antimicrobial resistance. Both the antibiotics may be preferred in treatment of bacterial mastitis as they achieve therapeutic concentration in milk for a sufficient period. However, ceftriaxone at a proper dosage regimen may replace use of ceftizoxime in goats and cows in which ceftriaxone is metabolized to ceftizoxime so that ceftizoxime could be reserved for treating severe human bacterial infections. Moreover, antibiotic sensitivity test for ceftriaxone against causative bacteria isolated from animals like goats, cows or other animal species in which ceftriaxone is metabolized to ceftizoxime should include ceftizoxime disc along with ceftriaxone disc.
